# Early Effects of a Low Fat, Fructose-Rich Diet on Liver Metabolism, Insulin Signaling, and Oxidative Stress in Young and Adult Rats

**DOI:** 10.3389/fphys.2018.00411

**Published:** 2018-04-26

**Authors:** Raffaella Crescenzo, Luisa Cigliano, Arianna Mazzoli, Rosa Cancelliere, Rosa Carotenuto, Margherita Tussellino, Giovanna Liverini, Susanna Iossa

**Affiliations:** Department of Biology, University of Naples Federico II, Naples, Italy

**Keywords:** fructose diet, young and adult rats, inflammation, insulin resistance, hepatic oxidative stress

## Abstract

The increase in the use of refined food, which is rich in fructose, is of particular concern in children and adolescents, since the total caloric intake and the prevalence of metabolic syndrome are increasing continuously in these populations. Nevertheless, the effects of high fructose diet have been mostly investigated in adults, by focusing on the effect of a long-term fructose intake. Notably, some reports evidenced that even short-term fructose intake exerts detrimental effects on metabolism. Therefore, the aim of this study was to compare the metabolic changes induced by the fructose-rich diet in rats of different age, i.e., young (30 days old) and adult (90 days old) rats. The fructose-rich diet increased whole body lipid content in adult, but not in young rats. The analysis of liver markers of inflammation suggests that different mechanisms depending on the age might be activated after the fructose-rich diet. In fact, a pro-inflammatory gene-expression analysis showed just a minor activation of macrophages in young rats compared to adult rats, while other markers of low-grade metabolic inflammation (TNF-alpha, myeloperoxidase, lipocalin, haptoglobin) significantly increased. Inflammation was associated with oxidative damage to hepatic lipids in young and adult rats, while increased levels of hepatic nitrotyrosine and ceramides were detected only in young rats. Interestingly, fructose-induced hepatic insulin resistance was evident in young but not in adult rats, while whole body insulin sensitivity decreased both in fructose-fed young and adult rats. Taken together, the present data indicate that young rats do not increase their body lipids but are exposed to metabolic perturbations, such as hepatic insulin resistance and hepatic oxidative stress, in line with the finding that increased fructose intake may be an important predictor of metabolic risk in young people, independently of weight status. These results indicate the need of corrective nutritional interventions for young people and adults as well for the prevention of fructose-induced metabolic alterations.

## Introduction

In the last decades, high fructose intake has been associated with increased risk of obesity development and the consequent metabolic and inflammatory diseases (Bray and Popkin, 2014; Campos and Tappy, 2016). Liver function is particularly vulnerable to an increasing fructose intake, since this organ is responsible for about 90% of the total metabolism of this sugar (Tappy and Lê, 2010). Accordingly, different studies in both human and animal models evidenced liver morphological and functional damage following high intake of fructose or fructose-containing sweeteners (Crescenzo et al., 2013, 2014, 2017; Jin and Vos, 2015). In addition, it has been recently reported that even perinatal exposure to maternal consumption of sucrose or high fructose corn syrup exerts detrimental effects on the offspring, particularly on adiposity, plasma non-esterified fatty acids (NEFA) and hepatic lipid content (Toop et al., 2017).

Most research studies have been focused on the long term effects of high fructose intake, but some results evidenced that even short-term high fructose intake could exert detrimental effects on metabolic state. Even 7 days of high fructose intake elicit an increase in liver triglycerides in humans (Sobrecases et al., 2010). Moreover, it has been shown that 2 weeks of high fructose diet in weaning rats have a prooxidant effect on different tissues (Busserolles et al., 2002). In addition, hepatic metabolic alterations and increased markers of oxidative stress and inflammation were detected after 3 weeks of fructose administration in drinking water in rats (Francini et al., 2010; Castro et al., 2012, 2013, 2014, 2015) or after 4 and 8 weeks of increased fructose in the diet (Alwahsh et al., 2014, 2017).

The increase in the use of refined food rich in fructose is of particular concern in children and adolescents, since the total caloric intake and the prevalence of metabolic syndrome are increasing continuously in these populations (Bremer and Lustig, 2012; Bremer et al., 2012). It has already been demonstrated that increased sugar-sweetened beverages intake is linked to the development of childhood obesity (Bray and Popkin, 2013) and may be an important predictor of metabolic risk in young people, independently of weight status (Ambrosini et al., 2013). Although there is an obvious need of clarifying age-specific metabolic effects of this sugar, most of the investigations on the deleterious effects of fructose overconsumption were undertaken in adults. Although young populations consume more fructose-rich drinks than adults (Ford et al., 2013), few reports addressed the metabolic effects of high fructose intake in young organisms (Araujo et al., 2016; Farah et al., 2016; Mock et al., 2017), and moreover these studies pointed their attention on the long-term effects of a fructose-rich diet, without addressing possible age-related differences.

Hence, a major aim of this study was to compare the metabolic alterations induced by the fructose-rich diet in rats of different age, i.e., young (30 days old) and adult (90 days old) rats. We have recently shown that this experimental design induces inflammation, oxidative stress, and mitochondrial impairment in rat hippocampus in an age-dependent fashion (Cigliano et al., 2018). In detail, at the end of the dietary treatment, metabolic characterization was performed by carrying out energy balance measurements, together with analysis of plasma lipids and glucose tolerance. In addition, liver insulin signaling, the degree of oxidative stress in both liver homogenates and mitochondria as well as markers of low-grade metabolic inflammation in plasma and liver were evaluated after fructose feeding in rats of different ages.

## Materials and methods

### Materials

Bovine serum albumin fraction V (BSA), o-phenylenediamine, hexadecyltrimethylammonium bromide (HTAB), 3-amino-1,2,4 triazole, as well as salts, buffers and reagents used in the biochemical analyses were purchased from Sigma-Aldrich (St. Louis, MO, USA). The dye reagent for protein titration was from Bio-Rad (Hercules, CA, USA). Polystyrene 96-wells ELISA MaxiSorp plates, with high affinity to proteins with mixed hydrophilic/hydrophobic domains, were purchased from Nunc (Roskilde, Denmark).

### Animals and treatments

All experimental procedures involving animals were approved by “Comitato Etico-Scientifico per la Sperimentazione Animale” of the University of Naples Federico II and authorized by Italian Health Minister (260/2015-PR). This work complies with the animal ethics principles and regulations of the Italian Health Ministry. The authors ensured that all steps were taken to minimize the pain and suffering of the animals.

Male Sprague-Dawley rats (Charles River, Italy), of 30 (young) or 90 (adult) days of age were caged singly in a temperature-controlled room (23 ± 1°C) with a 12-h light/dark cycle (06.30–18.30). Young and adult rats were divided in three groups: the first one was euthanized at the beginning of the dietary treatment to measure initial body composition, while the remaining two groups were fed a fructose-rich or control diet for 2 weeks. The composition of the two diets is shown in Table 1. Rats were pair-fed for the whole experimental period, by giving them the same amount of diet, both as weight and as caloric content and each rat consumed the full portion of the diet. The amount of daily caloric intake of all groups of rats steadily increased during the experimental period. During the treatment, body weight and food intake were monitored daily. At the end of the experimental period, the rats were euthanized by decapitation, and blood and liver were collected, while the carcasses were used for body composition determination.

**Table 1:** Composition of experimental diets.

	**Control diet**	**Fructose diet**
**COMPONENT (g/100 g)**		
Standard chow^*^	50.5	50.5
Sunflower oil	1.5	1.5
Casein	9.2	9.2
Alphacel	9.8	9.8
Starch	20.4	–
Fructose	–	20.4
Water	6.4	6.4
AIN-76 mineral mix	1.6	1.6
AIN-76 vitamin mix	0.4	0.4
Choline	0.1	0.1
Methionine	0.1	0.1
Gross energy density, kJ/g	17.2	17.2
Metabolisable energy density, kJ/g^**^	11.1	11.1
Protein, % metabolisable energy	29.0	29.0
Lipids, % metabolisable energy	10.6	10.6
Carbohydrates, % metabolisable energy	60.4	60.4
Of which: Fructose	–	30.0
Starch	52.8	22.8
Sugars	7.6	7.6

* *Mucedola 4RF21; Italy*.

** *Estimated by computation using values (kJ/g) for energy content as follows: protein 16.736, lipid 37.656, and carbohydrate 16.736*.

### Indirect calorimetry

Oxygen consumption and carbon dioxide production of the rats were recorded over a period of 24 h with a four-chamber indirect open-circuit calorimeter (Panlab s.r.l., Barcelona, Spain). Measurements were performed every 15 min for 3 min in each cage. Urine was collected for the whole (24-h) period and urinary nitrogen levels were measured by an enzymatic colorimetric method (FAR Diagnostics s.r.l., Settimo di Pescantina, Italy). Urinary nitrogen levels were then used to calculate oxygen consumption and carbon dioxide production due to protein oxidation, and the obtained values were subtracted from total oxygen consumption and carbon dioxide production to calculate non-protein oxygen consumption and carbon dioxide production. These latter values were finally used to obtain the non-protein respiratory quotient (NPRQ) values in the different rat groups.

### Glucose tolerance test

Glucose tolerance test was carried out the day before the euthanasia. To this end, rats were fasted for 6 h from 08.00 a.m. Basal, postabsorptive blood sample was obtained from a small tail clip and placed in EDTA–coated tubes and then glucose (2 g/kg body weight) was injected intraperitoneally. Blood samples were collected after 20, 40, 60, 90, and 120 min and placed in EDTA-coated tubes. The blood samples were centrifuged at 2000 × g for 15 min at 4°C. Plasma glucose concentration was measured by colorimetric enzymatic method (Pokler Italia, Genova, Italy), while plasma insulin concentration was measured using an ELISA kit (Mercodia AB, Uppsala, Sweden) in a single assay to remove inter-assay variations.

### Body composition and energy balance

Guts were cleaned of undigested food and the carcasses were then autoclaved. After dilution in distilled water and subsequent homogenization of the carcasses with a Polytron homogeniser (Kinematica, Switzerland), duplicate samples of the homogenized carcass were analyzed for energy content by bomb calorimeter. The contribution of the liver to total body energy content was taken into account by measuring energy content of liver samples with the bomb calorimeter. Total body water content was determined by drying carcass samples in an oven at 60°C for 48 h. Total body lipids were measured by the Folch extraction method (Folch et al., 1957). The energy as lipid was calculated from body lipids by using the coefficient of 39.2 kJ/g, and was then subtracted from total body energy to obtain the energy as protein. Energy balance measurements were conducted by the comparative carcass technique over the experimental period, as previously detailed (Crescenzo et al., 2010). Briefly, daily food consumption was monitored, energy density of the diet was measured by a bomb calorimeter and gross energy intake was calculated. Energy content was measured in feces and urine and the values were subtracted from gross energy intake to obtain metabolizable energy (ME) intake. Body energy, lipid and protein gain were calculated as the difference between the final and initial content of body energy, fat and protein. Energy expenditure was determined as the difference between energy gain and ME intake, and the energetic efficiency was calculated as the percentage of total energy gain per ME intake.

### Plasma metabolic parameters and liver composition

Plasma concentrations of alanine aminotransferase (ALT), triglycerides, and non esterified fatty acids (NEFA) were measured by colorimetric enzymatic method using commercial kits (SGM Italia, Rome, Italy for ALT and triglycerides and Roche Diagnostics, Mannheim, Germany for NEFA). Lipid peroxidation was determined according to Fernandes et al. (2006), by measuring thiobarbituric acid reactive substances (TBARS), using the thiobarbituric acid assay.

Liver triglycerides were measured by colorimetric enzymatic method using commercial kits (SGM Italia, Rome, Italy). Hepatic lipid content data were also confirmed by histological analysis. Frozen 10 μm-thick sections were obtained after embedding and freezing liver fragments in Killik (Bio Optica, Milan, Italy). Sections were then stained with hematoxylin-eosin and mounted in PBS/glycerol (9:1, v/v) (Tussellino et al., 2012; Alwahsh et al., 2014). Sections were photographed with a Leica CTR 6500 UV microscope (Leica Microsystems CMS gmbH, Wetzlar, Germany) and degree of steatosis was evaluated by ImageJ software.

Hepatic glycogen content was assessed by direct enzymatic procedure (Roehrig and Allred, 1974).

Liver ceramide content was evaluated by ELISA using 96-well Polysorp plates (Nunc, Rochester, NY, USA). Briefly, hepatic lipids extracted with the Folch method (Folch et al., 1957) were adsorbed to well bottoms overnight at 4°C. Plates were blocked with 10 mM phosphate buffered saline (PBS), 140 mM NaCl, 0.1% Tween, pH 7.4 supplemented with 1% bovine serum albumin for 1 h at 37°C. The plates were then washed three times with washing buffer containing 10 mM PBS, 140 mM NaCl, 0.05% Tween, pH 7.4 and incubated with monoclonal anti-ceramide antibody (2 μg/ml) for 1 h at 37°C. After three washings, peroxidase-conjugated goat anti-mouse IgM (1:5000 dilution) was incubated for 1 h at 37°C. After four washings, the wells were incubated with 100 μl of a color development solution (20 mg of o-Phenylenediamine dihydrochloride in 50 ml of 70 mM Na_2_HPO_4_, 30 mM citric acid, pH 5, supplemented with 120 μl of 3% H_2_O_2_). After 15 min at 37°C, the reaction was stopped by the addition of 50 μl of 2.5 M H_2_SO_4_ and the absorbance was measured at 492 nm. All tests were done in triplicate. Immunoreactivity was normalized to starting tissue weight. Negative control reactions were performed by omitting the addition of primary antibody.

### Markers of inflammation in liver and plasma

As plasma marker of inflammation, the concentration of the acute phase protein haptoglobin (Hpt) was measured by ELISA as previously reported (Spagnuolo et al., 2014). Individual samples were diluted (1:10,000–1:60,000) with coating buffer (7 mM Na_2_CO_3_, 17 mM NaHCO_3_, 1.5 mM NaN_3_, pH 9.6), and incubated in the wells of a microtitre plate (Immuno MaxiSorp; overnight, 4°C). After four washes by Tween-Tris buffered saline (T-TBS) (130 mM NaCl, 20 mM Tris-HCl, 0.05% Tween 20, pH 7.4) and four washes by high salt TBS (500 mM NaCl in 20 mM Tris-HCl at pH 7.4), the wells were blocked with TBS (130 mM NaCl, 20 mM Tris-HCl, pH 7.4) containing 0.5% BSA (1 h, 37°C). After washing, the wells were incubated (1 h, 37°C) with 60 μl of rabbit anti-Hpt IgG (ICL Lab, 1:1000 dilution) followed by goat anti-rabbit peroxidase-conjugated secondary antibody (GAR-HRP IgG; 1:8,000 dilution; Sigma-Aldrich, MO, USA) for Hpt detection. Peroxidase-catalyzed color development from *o*-phenylenediamine was measured at 492 nm.

Tumor necrosis factor (TNF)-alpha concentrations in protein extracts from liver were determined using a rat specific enzyme linked immunosorbent assay (ELISA) (R&D Systems, Minneapolis, MN, USA) according to manufacturer's instruction.

The determination of myeloperoxidase (MPO) activity can be used as a surrogate marker of inflammation, since it has been shown that the activity of MPO solubilized from the inflamed tissue is directly proportional to the number of neutrophils seen in histologic sections (Krawisz et al., 1984). MPO activity was therefore assessed in liver samples as reported by Kim et al. (2012). Briefly, tissue samples (100 mg) were homogenized in 1 ml of HTAB buffer (0.5% HTAB in 50 mM phosphate buffer, pH 6.0) and centrifuged at 13,400 × g for 6 min at 4°C. Then, 10 μl of supernatant were combined with 200 μl of 50 mM phosphate buffer, pH 6.0, containing 0.167 mg/ml 0-dianisidine hydrochloride and 1.25% hydrogen peroxide. The change in absorbance at 450 nm was measured and one unit of MPO activity was defined as that degrading 1 μmol of peroxide per minute at 25°C.

For Real-Time RT-PCR analysis of inflammation markers in liver, total RNA was extracted from single liver for sample randomly selected for each treatment using Tri-Reagent (Sigma, St. Louis, MO, USA) according to the manufacturer's recommendations. cDNAs were obtained, from 1 μg of total RNA, using the Super Script VILO cDNA synthesis kit (Life Technologies, Thermo Fisher Scientific, Waltham, MA, USA) following the manufacturer's instructions. Real-time PCR was performed using Power SYBR Green Master Mix kits (Life Technologies, Thermo Fisher Scientific, Waltham, MA, USA) using the 96-well optical reaction plate in 20 μL total reaction volume. The reaction solution and amplification reactions were carried out as reported in De Marco et al. (2017) and Tussellino et al. (2016), respectively. β-actin (Alwahsh et al., 2014) was designed as endogenous reference (housekeeping gene). cDNA from samples of adult rat were used as controls. Primers used to detect Monocyte Chemoattractant Protein 1 (MCP1) (Sheldon et al., 2015), Arginase (*Arg*) (Li et al., 2012), F4/80 (Sheldon et al., 2015), and Interleukin 1 beta (*IL-1*β*)* (Sheldon et al., 2015) are shown in Table 2.

**Table 2:** Primers used for real-time RT-PCR analysis.

**Gene name**	**Oligo forward sequence**	**Oligo reverse sequence**
MCP-1	CTG TCT CAG CCA GAT GCA GTT AA	AGC CGA CTC ATT GGG ATC AT
F4/80	GGA GGA CCA ATG TTC CAG GG	TGG GCA AGA ACA GCT GTA GG
IL-1beta (*IL-1β*)	CCT ATG TCT TGC CCG TGG AG	CAC ACA CTA GCA GGT CGT CA
Arginase-1 (*Arg*)	AAG AAA AGG CCG ATT CAC CT	CAC CTC CTC TGC TGT CTT CC
β-actin (*Actb*)	ACC ACC ATG TAC CCA GGC ATT	CCA CAC AGA GTA CTT GCG CTC A

### Isolation of liver mitochondria and measurement of mitochondrial oxidative capacities

Isolation of liver mitochondria and measurement of state 3 respiration were carried out as previously reported (Crescenzo et al., 2012). Briefly, liver tissue fragments were gently homogenized with a medium containing 220 mM mannitol, 70 mM sucrose, 20 mM Hepes, 1 mM EDTA, and 0.1% (w/v) fatty acid free BSA, pH 7.4, in a Potter Elvehjem homogenizer set at 500 rpm (4 strokes/min). After withdrawn of aliquots for further assays, the homogenate was then centrifuged at 1,000 g for 10 min and the resulting supernatant was again centrifuged at 3000 g for 10 min. The mitochondrial pellet was washed twice and finally resuspended in a medium containing 80 mM KCl, 50 mM HEPES, 5 mM K_2_HPO_4_, 1 mM EGTA, 0.1% (w/v) fatty acid-free BSA, pH 7.0. Oxygen consumption rate was measured polarographically in liver homogenates and isolated mitochondria with a Clark-type electrode (Yellow Springs Instruments, Yellow Springs, OH, USA) in a 3 ml-glass cell, at a temperature of 30°C in a medium containing 80 mM KCl, 50 mM HEPES, 5 mM K_2_HPO_4_, 1 mM EGTA, 0.1% (w/v) fatty acid-free BSA, pH 7.0. All samples were allowed to oxidize their endogenous substrates for 3 min and then 10 mM glutamate + 2.5 mM malate, 10 mM succinate + 3.8 μM rotenone, or 40 μM palmitoyl-carnitine + 2.5 mM malate were added as substrate. State 3 oxygen consumption was measured in the presence of 0.3 mM ADP. Control experiments of enzymatic and electron microscopy characterization have shown that our isolation procedure (centrifugation at 3, 000 × g for 10 min) results in a cellular fraction, which is constituted essentially by mitochondria.

### Hepatic *de novo* lipogenesis, mitochondrial lipid peroxidation, titration of nitro-tyrosine (N-Tyr) and manganese-superoxide dismutase (Mn-SOD) specific activity

De novo lipogenesis in liver was evaluated by assessing fatty acid synthase (FAS) activity, according to the protocol described by Pénicaud et al. (1991) on hepatic homogenates.

Lipid peroxidation was determined in liver homogenates and isolated mitochondria by using the same procedure used for plasma samples as reported above.

As marker of protein oxidative modifications, the level of N-Tyr in liver homogenates and isolated mitochondria was quantified by ELISA. Samples were diluted (1:500, 1:1,500, 1:3,000, and 1:6,000) with coating buffer, and aliquots (50 μl) were then incubated in the wells of a microtitre plate (overnight, 4°C). After four washes by T-TBS and four washes by high salt TBS, the wells were blocked with TBS containing 0.5% BSA (1 h, 37°C). After washing, the wells were incubated (2 h, 37°C) with 50 μl of rabbit anti-N-Tyr (Covalab, purchased by Vinci Biochem, Vinci, Italy; 1: 800 dilution in T-TBS containing 0.25% BSA) followed by 60 μl of GAR-HRP IgG (1:4,000 dilution; 1 h, 37°C). Peroxidase-catalyzed color development from *o*-phenylenediamine was measured at 492 nm. Data were reported as OD per mg of proteins.

Mn-SOD specific activity was measured in liver mitochondria in a medium containing 0.1 mM EDTA, 2 mM KCN, 50 mM KH2PO4 pH 7.8, 20 mM cythocrome c, 0.1 mM xanthine, and 0.01 units of xanthine oxidase. Determinations were carried out spectrophotometrically (550 nm) at 25°C, by monitoring the decrease in the reduction rate of cytochrome c by superoxide radicals, generated by the xanthine-xanthine oxidase system. One unit of SOD activity is defined as the concentration of enzyme that inhibits cytochrome c reduction by 50% in the presence of xanthine+xanthine oxidase (Flohè and Otting, 1974).

### Quantification of activation of insulin receptor substrate 1 (IRS1), protein kinase b (Akt) and lipocalin 2 (LCN-2)

Proteins were extracted from livers by homogenizing frozen tissues (−80°C) in 10 volumes (w/v) of lysis buffer containing 20 mM Tris-HCl (pH 7.5), 150 mM NaCl, 2.7 mM KCl, 5% (v/v) glycerol, 1% (v/v) Triton X-100 and 50 μL/g tissue of protease inhibitor cocktail (all from Sigma-Aldrich, St. Louis, MO, USA) using a Potter homogeniser, shaken for 2 h at 4°C. Homogenates were centrifuged at 14,000 g_av_ for 20 min at 4°C and the supernatants were collected. Then, aliquots (65 μg) were denatured in Laemmli's buffer (60.0 mM Tris pH 6.8, 10% sucrose, 2% SDS, 4% β-mercaptoethanol) and fractionated by electrophoresis under denaturing and reducing conditions on 10% (for p-IRS and p-Akt) or 12% (for LCN-2) polyacrylamide gel. After the run in electrode buffer (25 mM Tris, pH 8.3, 192 mM glycine, 0.1% SDS), the gels were transferred onto polyvinylidene difluoride membranes (PVDF, Immobilon-P, Merck Millipore, Darmstadt, Germany) at 0.8 mA/cm^2^ for 90 min.

For quantification of p-IRS, the membranes were blocked in TBS containing 3% BSA and 0.1% Tween for 1 h and then incubated overnight at 4°C with rabbit anti-pIRS1 (Tyr612) (Thermo Fisher Scientific, Waltham, MA, USA, 1:500 dilution in blocking buffer). Membranes were washed 3 times 12 min in TBS/0.1% Tween 20 and 3 times 12 min in TBS and then incubated with GAR-HRP IgG (1: 7,000 dilution in blocking buffer; 1 h, 37°C) (Sigma-Aldrich, St. Louis, MO, USA). The membranes were washed as above described, rinsed in distilled water and the immunocomplexes were detected by the ECL detection system (Immobilon Western Chemiluminescent HRP kit, distributed by Microtech, Naples, Italy). After p-IRS detection, the membrane was extensively washed with TBS/0.1% Tween 20, and submerged in stripping buffer (100 mM β-mercaptoethanol, 2% SDS, 62.5 mM Tris-HCl, pH 6.7; 45 min, 50°C). Total IRS 1 was revealed by incubation (overnight, 4°C) with rabbit anti-IRS1 (Cell Signaling, Danvers, MA, USA, 1:500 dilution in blocking buffer) followed by GAR-HRP IgG (1: 20,000 dilution in blocking buffer; 1 h, 37°C).

For quantification of p-Akt, the membranes were blocked in blocking buffer (TBS; 5% milk powder; 0.1% Tween 20) for 1 h and then incubated overnight at 4°C with rabbit anti-p-Akt (Cell Signaling, Danvers, MA, USA, diluted 1:2,000 in blocking buffer). Membranes were washed 3 times 15 min in TBS/0.1% Tween 20 and 3 times 15 min in TBS and then incubated 1 h at room temperature with an anti-rabbit alkaline phosphatase-conjugated secondary antibody (GAR-AP IgG; diluted 1:5,000 in blocking buffer; 1 h, 37°C) (Promega, Madison, WI, USA). The membranes were washed as above described, rinsed in distilled water and incubated at room temperature with a chemiluminescent substrate, CDP-Star (Sigma-Aldrich, St Louis, MO, USA). After p-Akt detection, the membrane was stripped as above and incubated overnight at 4°C with rabbit anti-Akt (Cell Signaling, Danvers, MA, USA, 1:2000 dilution in blocking buffer), followed by an anti-rabbit alkaline phosphatase-conjugated secondary antibody (GAR-AP IgG; diluted 1:5,000 in blocking buffer; 1 h, 37°C) (Promega, Madison, WI, USA).

For quantification of LCN-2, the membranes were blocked in blocking buffer (TBS; 5% milk powder; 0.1% Tween 20) for 1 h, 37°C and then incubated overnight at 4°C with goat anti- LCN-2 (Thermo Fischer Scientific, Waltham, MA, USA; 1:300 dilution in blocking buffer containing 0.5% milk powder). Membranes were washed 3 times 15 min in TBS/0.1% Tween 20 and 3 times 15 min in TBS and then incubated 1 h at room temperature with a rabbit anti-goat peroxidase-conjugated secondary antibody (RAG-HRP IgG, 1:130,000 dilution in blocking buffer; 1 h, 37°C) (Sigma-Aldrich, St. Louis, MO, USA). The membranes were washed as above described, rinsed in distilled water and incubated at room temperature with the Excellent Chemiluminescent detection Kit (ElabScience, distributed by Microtech, Naples, Italy).

Rat plasma samples (75 μg protein) were denatured at 100°C for 5 min in Laemmli's buffer and fractionated by electrophoresis on 12% polyacrylamide gel, under denaturing and reducing conditions. After protein transfer onto PVDF as above described, the membranes were incubated with the same dilution of goat anti-LCN-2 and GAR-HRP IgG used for liver homogenates.

Data detection was carried out by exposing autoradiography films (Eastman Kodak Company, Long Island city, NY, USA) to the membranes and quantification of signals was carried out by Un-Scan-It gel software (Silk Scientific, Orem, UT, USA).

### Statistical analysis

Data were expressed as mean values ± SEM. The program GraphPad Prism 6 (GraphPad Software, San Diego, CA, USA) was used to verify that raw data have normal distribution and to perform two-tailed, two-way ANOVA followed by the Tukey *post-hoc* test. A probability of < 5% (*P* < 0.05) was considered statistically significant in all analyses.

## Results

### Body composition, energy balance, and NPRQ

**Figure 1:**
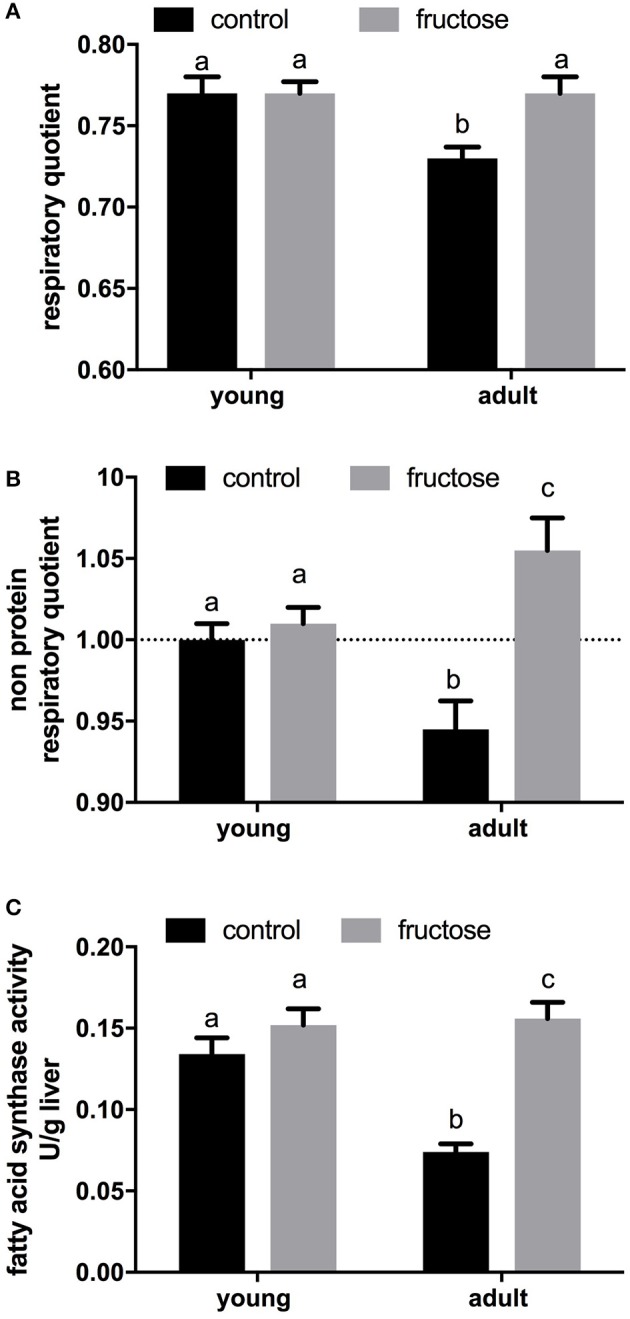
Indirect calorimetry measurements were performed on young and adult rats fed a control or fructose-rich diet for 2 weeks over a 24-h period and values of oxygen consumption, carbon dioxide production and urinary nitrogen were used to obtain respiratory quotient **(A)** and non-protein respiratory quotient values **(B)**. Fatty acid synthase activity **(C)** was measured in liver homogenates. Values are the means ± SEM of six different rats. Values with different superscript letters are significantly different (*P* < 0.05, Tukey post-test). Two-way ANOVA p results: **(A)** diet effect = 0.0312, age effect = 0.0312; **(B)** diet effect = 0.0007, age effect = 0.7430; **(C)** diet effect < 0.0001, age effect = 0.0056.

After 2 weeks of dietary treatment, body composition was evaluated. Even after this short term dietary treatment, the isoenergetic intake of a fructose-rich diet elicited a significant increase in body energy, body lipids, body energy gain, body lipid gain in adult rats, but not in young rats (Table 3). A trend toward increase was also found in young rats, but the relative variations were less evident and did not reach statistical significance. At the end of the dietary treatment, indirect calorimetry measurements were performed and values of oxygen consumption, carbon dioxide production, and urinary nitrogen were used to calculate energy expenditure per lean body mass, RQ and NPRQ. No significant difference in energy expenditure/g body protein was evident at the end of the dietary treatment and between age groups. On the other hand, a significant increase in RQ and NPRQ was found in adult but not in young rats after high fructose feeding (Figures 1A,B), thus indicating that after 2 weeks of high fructose intake the utilization of carbohydrates as metabolic fuels is increased. Part of this increase is due to the activation of *de novo* lipogenesis, as evidenced by the finding of increased FAS activity in the livers of adult rats (Figure 1C). No significant increase in FAS activity was found in young rats.

**Table 3:** Body composition and energy balance in young and adult rats fed a control or fructose-rich diet for 2 weeks.

	**Young**		**Adult**		**Two-way ANOVA** ***p*****-values**	
	**Control**	**Fructose**	**Control**	**Fructose**	**Diet effect**	**Age effect**
Initial body weight, g	100 ± 2^a^	100 ± 1^a^	500 ± 10^b^	500 ± 11^b^	>0.9999	< 0.0001
Final body weight, g	215 ± 4^a^	223 ± 3^a^	547 ± 11^b^	553 ± 16^b^	0.4931	< 0.0001
Body lipids, %	7.7 ± 0.3^a^	8.3 ± 0.4^a^	8.4 ± 0.3^a^	9.9 ± 0.3^b^	0.0045	0.0022
Body proteins, %	18.8 ± 0.9^a^	19.1 ± 1.0^a^	18.2 ± 0.7^a^	18.4 ± 0.8^a^	0.7736	0.4572
Body water, %	66.6 ± 0.4^a^	67.0 ± 0.7^a^	67.6 ± 0.5^a^	65.8 ± 0.7^a^	0.2490	0.8670
Body energy, kJ/g	7.4 ± 0.2^a^	7.7 ± 0.2^a^	7.6 ± 0.1^a^	8.5 ± 0.2^b^	0.0034	0.0117
Body weight gain, g^†^	115 ± 3^a^	123 ± 3^a^	47 ± 6^b^	53 ± 9^b^	0.2423	< 0.0001
Mean daily gross energy intake, kJ/day	448 ± 21^a^	437 ± 15^a^	614 ± 18^b^	625 ± 27^b^	>0.9999	< 0.0001
Body energy gain, kJ^†^	894 ± 66^a^	1050 ± 73^a^	487 ± 80^b^	1059 ± 62^a^	< 0.0001	0.0106
Body lipid gain, kJ^†^	374 ± 30^a^	463 ± 30^a^	175 ± 15^b^	541 ± 30^a^	< 0.0001	0.0368
Body protein gain, kJ^†^	523 ± 55^a^	589 ± 45^a^	395 ± 50^a^	440 ± 82^a^	0.2701	0.0103
ME intake, kJ^†^	4644 ± 292^a^	4582 ± 192^a^	6621 ± 238^b^	6759 ± 288^b^	0.8834	< 0.0001
Energy expenditure, kJ^†^	3750 ± 24^a^	3532 ± 177^a^	6134 ± 177^b^	5700 ± 163^b^	0.1049	< 0.0001
ME intake, kJ/g final body protein	115 ± 7^a^	108 ± 8^a^	66 ± 3^b^	66 ± 3^b^	0.5477	< 0.0001
Energy expenditure, kJ/g body protein	2.6 ± 0.2^a^	2.6 ± 0.2^a^	2.5 ± 0.2^a^	2.5 ± 0.2^a^	>0.9999	0.6225
Energetic efficiency, %	19.3 ± 0.8^a^	23.0 ± 0.9^b^	7.2 ± 0.5^c^	15.2 ± 0.7^d^	< 0.0001	< 0.0001

*Values are the means ± SEM of six different rats*.

† *Values refer to the whole period of the diet treatment (2 weeks). Values in the same row bearing different superscript letters are significantly different (p < 0.05, Tukey post-test). ME, metabolisable energy*.

### Analysis of glucose homeostasis and hepatic insulin signaling markers

**Figure 2:**
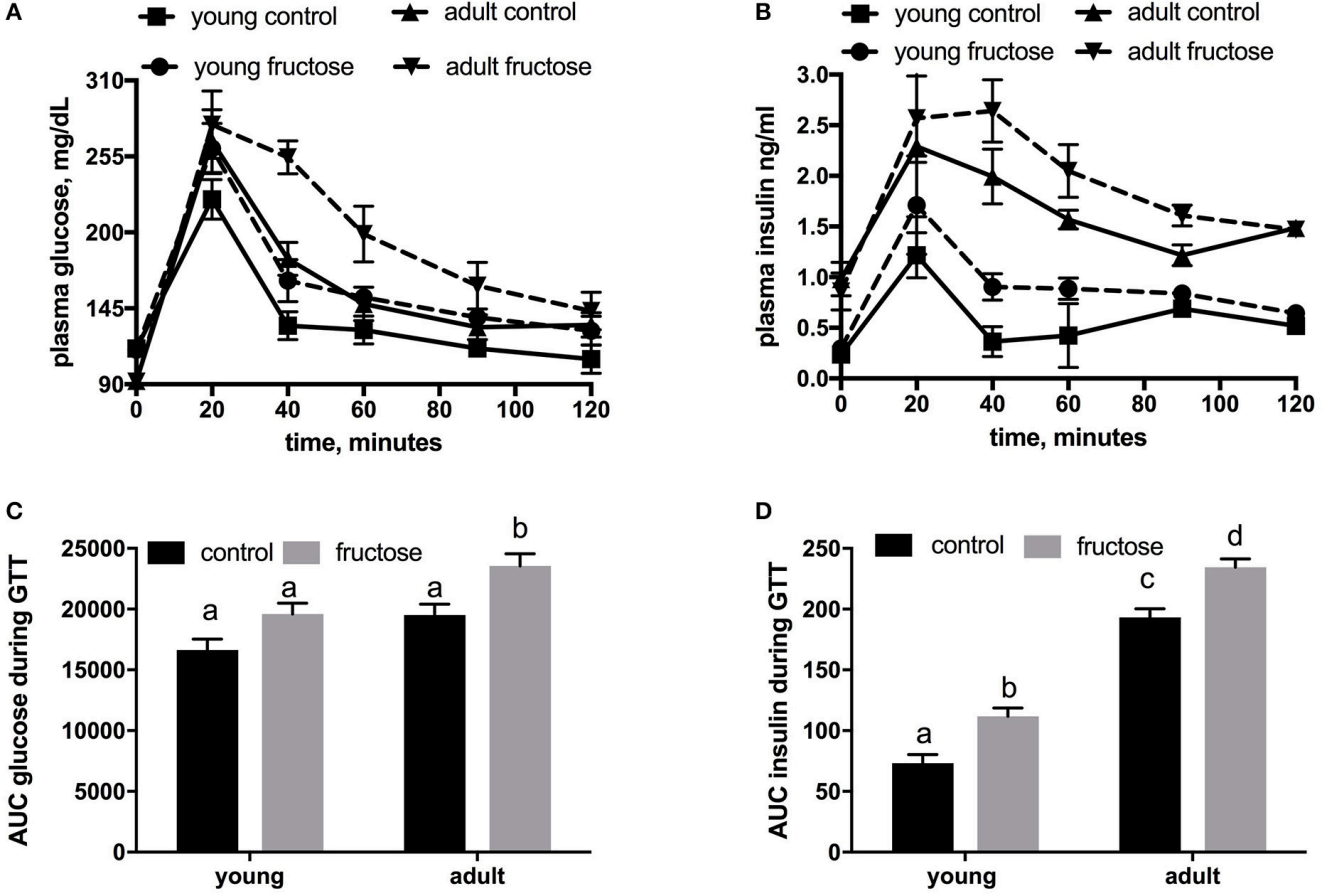
Whole body glucose homeostasis in young and adult rats fed a control or fructose-rich diet for 2 weeks. The day before the euthanasia, rats were fasted for 6 h, fasting blood samples were collected and then rats received an intraperitoneal injection of glucose (2 g/kg b.w.). Aliquots of blood were taken at 20, 40, 60, 90, and 120 min after injection and used for the determination of plasma glucose **(A)** and insulin **(B)** concentration. The area under the curve (AUC) of plasma glucose **(C)** and insulin **(D)** during glucose load was calculated with the trapezoid method. Values are the means ± SEM of six different rats. Values with different superscript letters are significantly different (*P* < 0.05, Tukey post-test). Two-way ANOVA p results: **(C)** diet effect = 0.0012, age effect = 0.00015; **(D)** diet effect < 0.0001, age effect < 0.0001.

**Figure 3:**
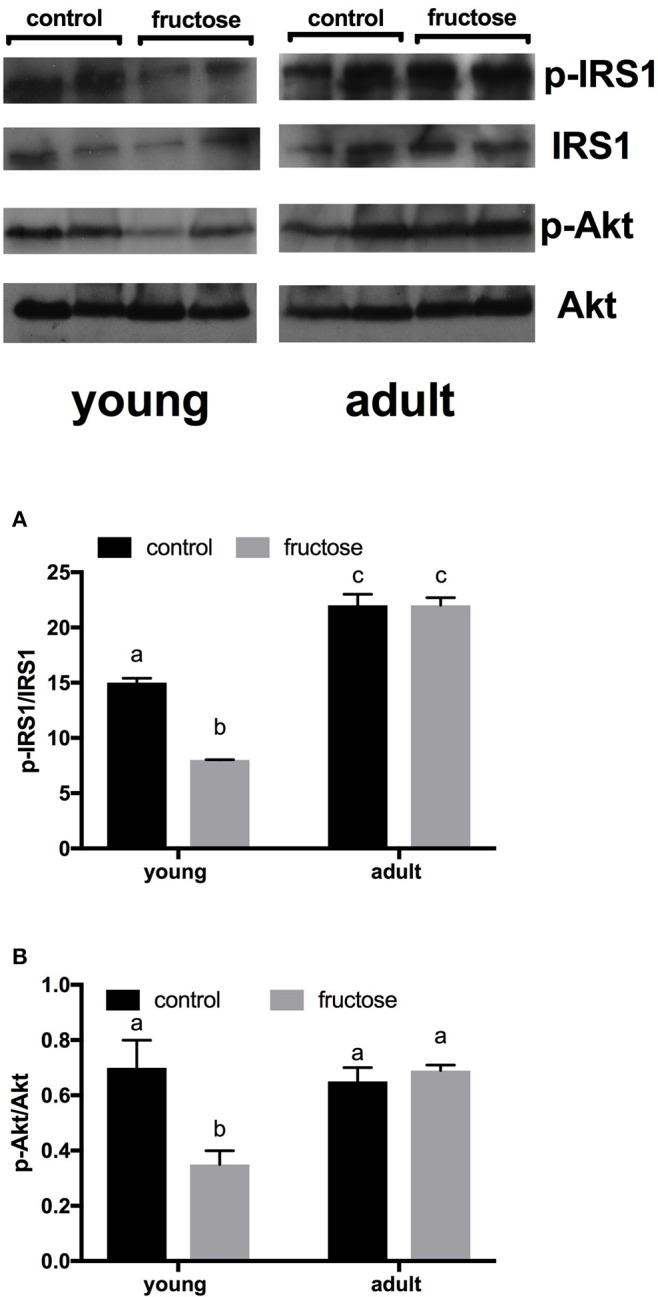
Hepatic degree of phosphorylation of insulin receptor substrate (IRS) 1 and protein kinase B (Akt), (**A**, **B** with representative western blots), evaluated by measuring p-IRS1/IRS1 ratio and p-Akt/Akt, respectively, by western blot on protein extracts from livers of young or adult rats fed a control or fructose-rich diet for 2 weeks. Data are reported as means ± SEM of six different rats. Values with different superscript letters are significantly different (*P* < 0.05, Tukey post-test). Two-way ANOVA p results: **(A)** diet effect < 0.0001, age effect < 0.0001; **(B)** diet effect = 0.0007, age effect < 0.0001.

Metabolic characterization of the glucose homeostatic system was obtained by carrying out glucose tolerance test the day before the euthanasia (Figures 2A,B). As shown in Figure 2C, area under the curve of plasma glucose was significantly higher in fructose-fed adult rats and tended to be higher in fructose-fed young ones. In addition, area under the curve of plasma insulin was significantly higher in adult than in young rats whatever the dietary treatment and was significantly higher in both young and adult rats fed a fructose-rich diet compared to controls (Figure 2D). Insulin signaling pathway in the liver was assessed through the degree of activation (by phosphorylation) of downstream targets, namely IRS1 and Akt. Significantly lower phosphorylation of both proteins was found in young rats after 2 weeks of fructose feeding (Figures 3A,B), while no significant effect was detected in the liver of adult rats (Figures 3A,B).

### Metabolites and markers of inflammation in plasma and liver

**Figure 4:**
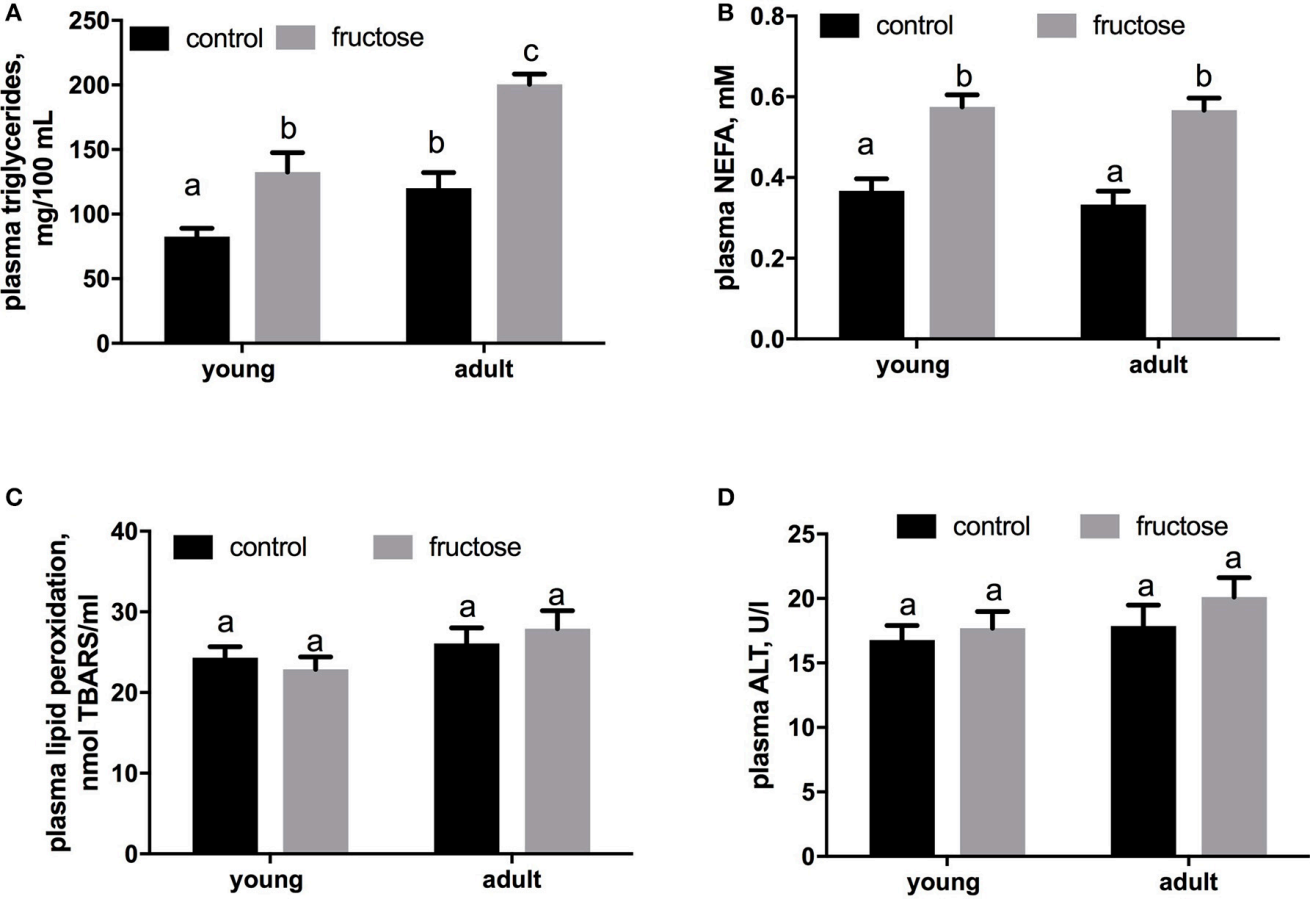
Plasma levels of triglycerides **(A)**, non-esterified fatty acids (NEFA) **(B)**, thiobarbituric acid reactive substances (TBARS) **(C)** and alanine aminotransferase (ALT) **(D)** in young and adult rats fed a control or fructose-rich diet for 2 weeks. Values are the means ± SEM of six different rats. Values with different superscript letters are significantly different (*P* < 0.05, Tukey post-test). Two-way ANOVA p results: **(A)** diet effect < 0.0001, age effect = 0.0002; **(B)** diet effect < 0.0001, age effect = 0.4587; **(C)** diet effect = 0.5879, age effect = 0.4557; **(D)** diet effect = 0.6621, age effect = 0.3587.

**Figure 5:**
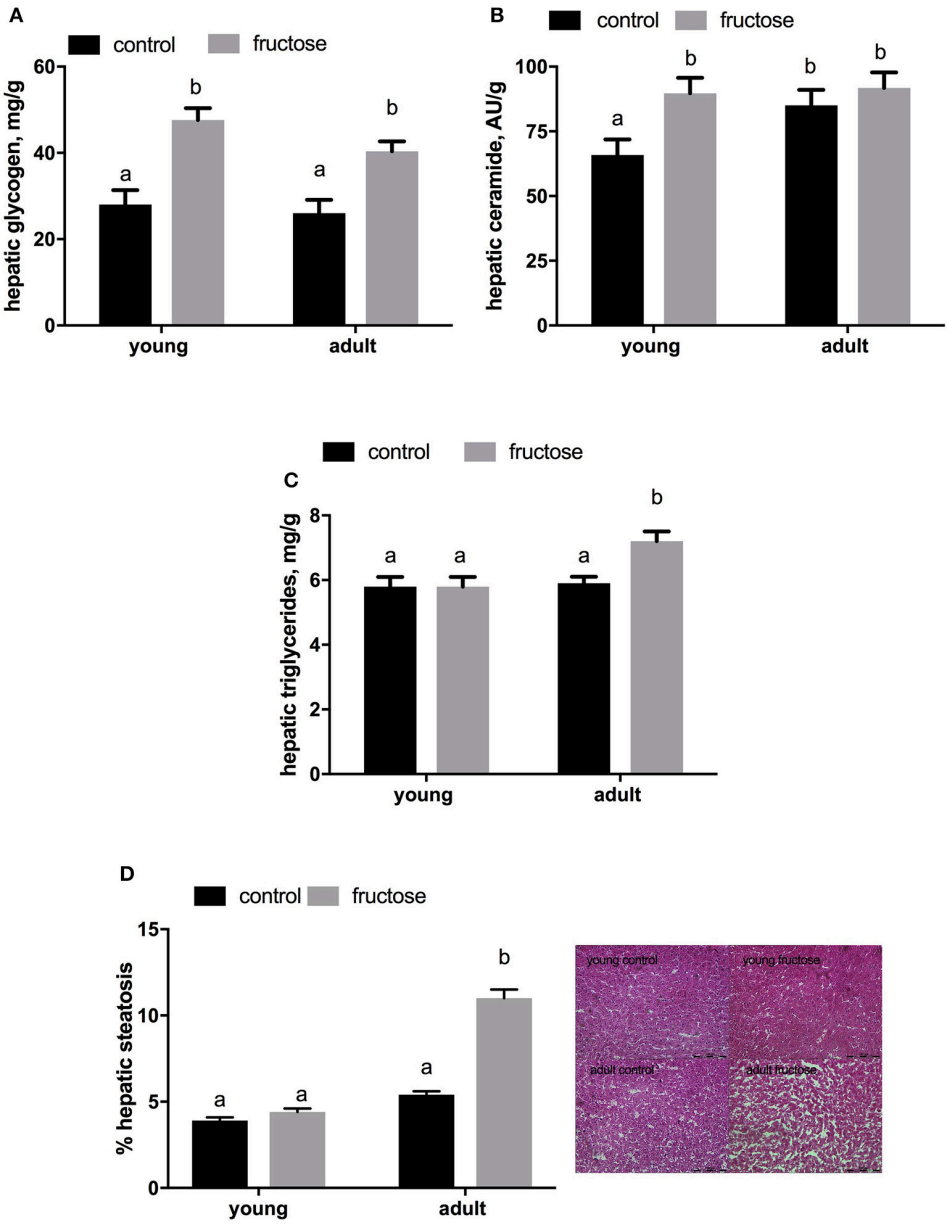
Hepatic content of glycogen **(A)** ceramide **(B)** and triglyceride (**C,D** with representative photographs) in young and adult rats fed a control or fructose-rich diet for 2 weeks. Values are the means ± SEM of six different rats. Values with different superscript letters are significantly different (*P* < 0.05, Tukey post-test). Two-way ANOVA p results: **(A)** diet effect < 0.0001, age effect = 0.2665; **(B)** diet effect = 0.0301, age effect = 0.014; **(C)** diet effect = 0.0076, age effect = 0.1917; **(D)** diet effect < 0.0001, age effect < 0.0001.

Plasma metabolites were assessed in young and adult rats fed a control or fructose-rich diet. A significant increase in plasma triglycerides (Figure 4A) and NEFA (Figure 4B) independent of age was elicited by fructose-rich diet, while no variation was found in plasma TBARS (Figure 4C) and ALT (Figure 4D). Liver glycogen content increased with fructose-rich diet in young and adult rats (Figure 5A). Fructose-rich diet significantly increased liver ceramide content only in young rats (Figure 5B), and liver content of triglycerides only in adult rats (Figures 5C,D).

**Figure 6:**
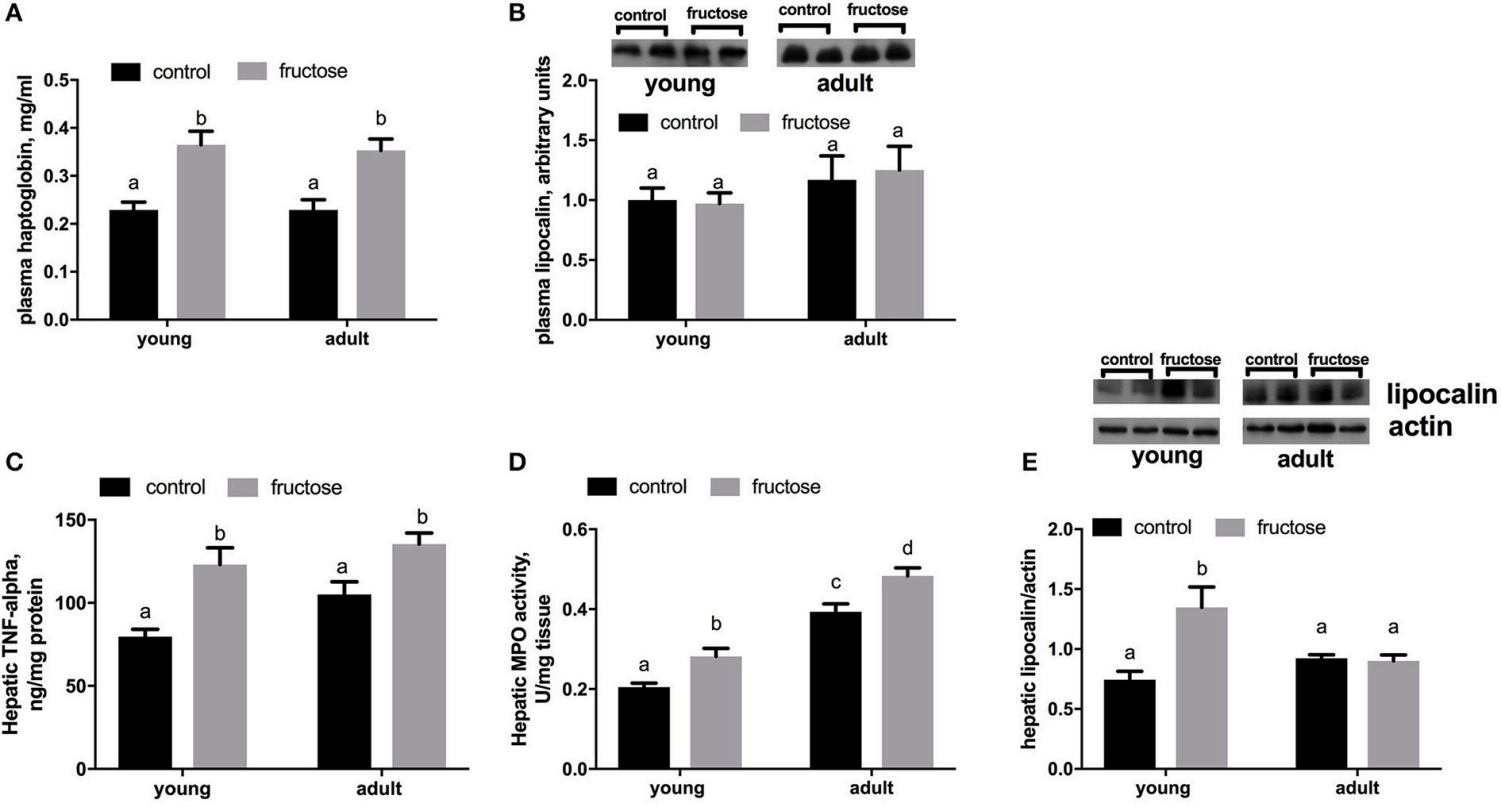
Haptoglobin **(A)** and lipocalin **(B)** in plasma samples, together with Tumor necrosis factor (TNF) alpha **(C)**, activity of the enzyme mieloperoxidase (MPO) **(D)** and lipocalin **(E)** in liver samples in young and adult rats fed a control or fructose-rich diet for 2 weeks. Lipocalin was assessed by western blot and representative blots are shown. For plasma lipocalin, equal loading was evaluated by Ponceau staining (data not shown). Values are the means ± SEM of six different rats. Values with different superscript letters are significantly different (*P* < 0.05, Tukey post-test). Two-way ANOVA p results: **(A)** diet effect = 0.0001, age effect = 0.7940; **(B)** diet effect = 0.8748, age effect = 0.1663; **(C)** diet effect < 0.0001, age effect = 0.0611; **(D)** diet effect = 0.0002, age effect < 0.0001; **(E)** diet effect = 0.0069, age effect = 0.1815.

As markers of inflammation we measured both Hpt and LCN-2 in plasma samples. The level of Hpt was found significantly higher in young and adult rats fed a fructose-rich diet (Figure 6A), while no variation was detected in plasma levels of LCN-2 (Figure 6B). Further, TNF-alpha protein levels and MPO activity were higher in rats fed fructose-rich diet regardless of age (Figures 6C,D). On the other hand, the amount of hepatic LCN-2 was found significantly increased by the fructose-rich diet only in young but not in adults (Figure 6E).

**Figure 7:**
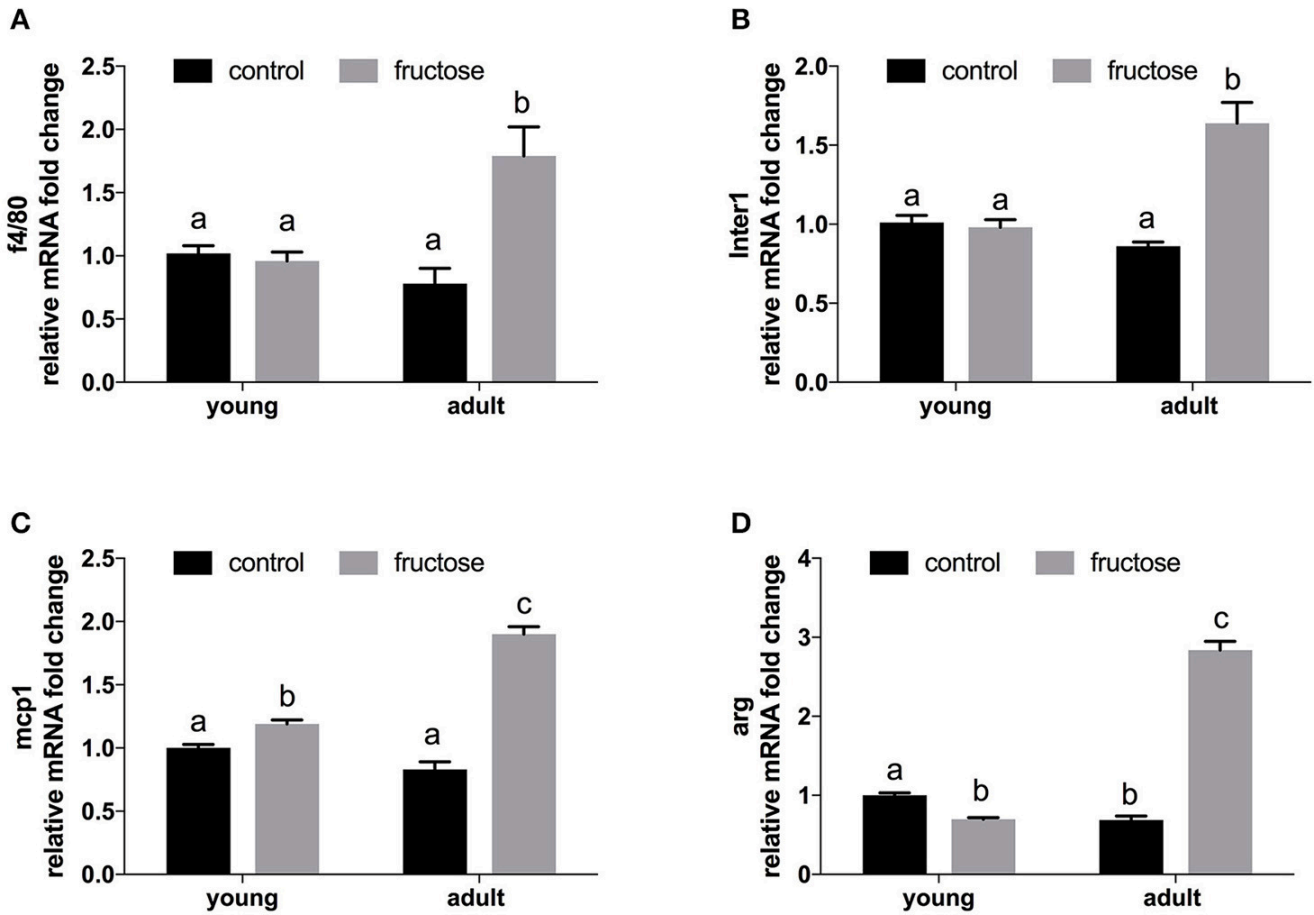
Relative mRNA fold change of markers of inflammation in liver samples from young and adult rats fed a control or fructose-rich diet for 2 weeks. **(A)** f4/80; **(B)** Inter1 = Interleukin 1; **(C)** mcp1 = Monocyte attractant protein 1; **(D)** Arg = Arginase. Values with different superscript letters are significantly different (*P* < 0.05, Tukey post test). Two-way ANOVA p results: **(A)** diet effect = 0.0039, age effect = 0.0608; **(B)** diet effect < 0.0001; age effect = 0.0017; **(C)** diet effect < 0.0001, age effect < 0.0001; **(D)** diet effect < 0.0001, age effect < 0.0001.

Liver gene expression profiling of inflammatory molecules, namely marker of macrophages (F4/80), of classical (MCP-1, IL-1), and alternative (Arg) macrophage activation revealed a marked upregulation of all the above markers in adult rats fed a fructose-rich diet (Figures 7A–D), while in young rats only slight changes (upregulation in MCP-1 and downregulation in Arg) were evident (Figure 7).

### Oxidative damage in liver homogenates and mitochondria

**Figure 8:**
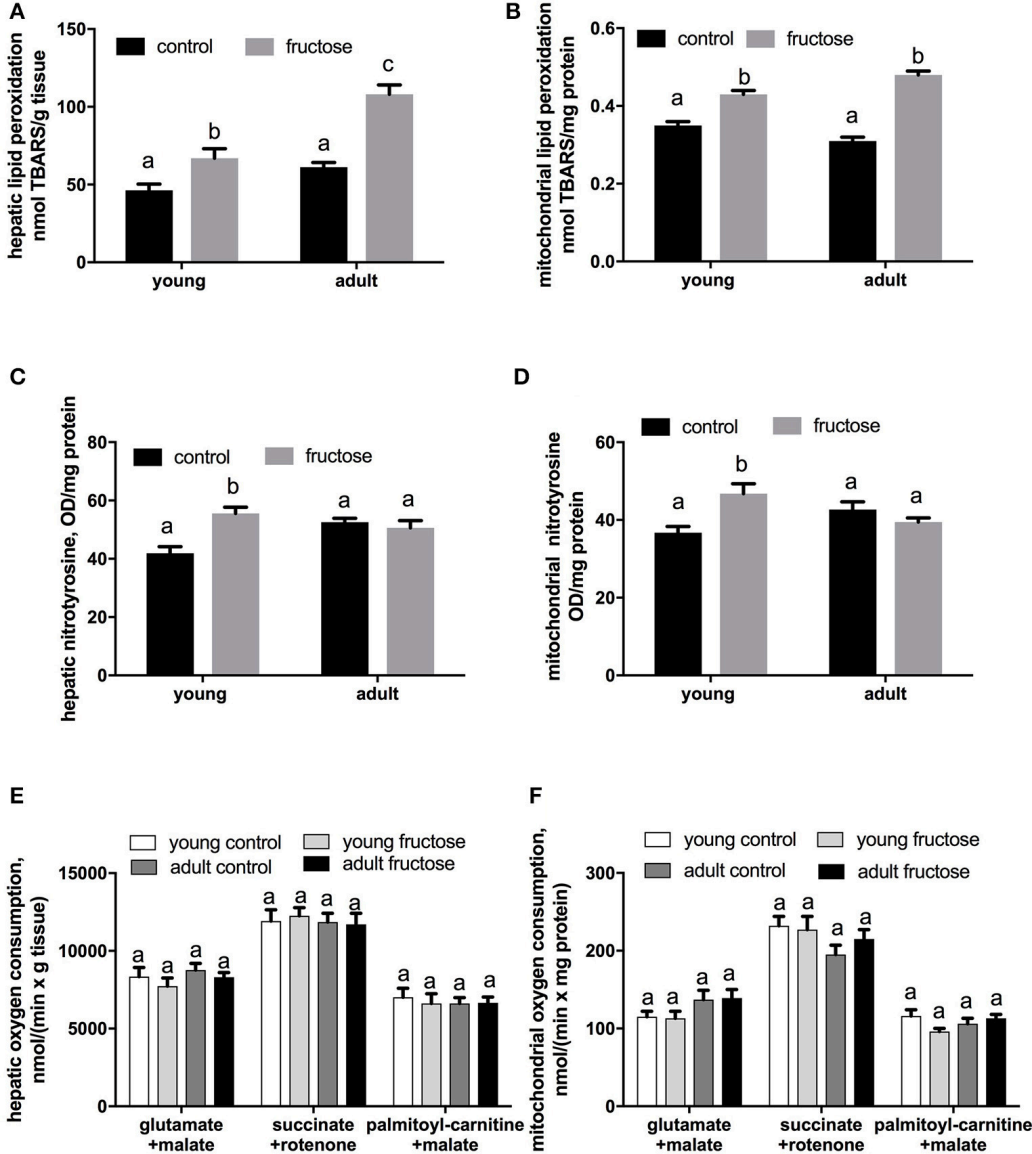
Oxidative damage to lipids in liver homogenates **(A)** and liver mitochondria **(B)**, nitrotyrosine content in liver homogenates **(C)**, and liver mitochondria **(D)**, and mitochondrial oxygen consumption measured in liver homogenates **(E)** and liver mitochondria **(F)** in young and adult rats fed a control or fructose-rich diet for 2 weeks. Measurements of oxygen consumption were carried out in the presence of saturating ADP (State 3) and using NAD-linked (glutamate+malate), FAD-linked (succinate+rotenone) or lipid (palmitoyl-carnitine+malate) substrate. Values are the means ± SEM of six different rats. Values with different superscript letters are significantly different (*P* < 0.05, Tukey post-test). Two-way ANOVA p results: **(A)** diet effect < 0.0001, age effect < 0.0001; **(B)** diet effect < 0.0001, age effect = 0.6225; **(C)** diet effect = 0.0138, age effect = 0.045; **(D)** diet effect = 0.035, age effect = 0.7267; **(E)** diet effect = 0.3522, age effect = 0.2726; **(F)** diet effect = 0.3455, age effect = 0.2967.

Oxidative damage to hepatic lipids was higher both in the whole tissue and at the mitochondrial level in fructose-fed rats (Figures 8A,B), while N-Tyr level, used as marker of protein oxidative modification, was found significantly increased only in fructose fed young rats both in the whole tissue and at the mitochondrial level (Figures 8C,D). No variation was found in mitochondrial oxidative capacities (Figures 8E,F) and in Mn-SOD specific activity in both groups of rats (data not shown).

## Discussion

In this work, we focused for the first time on the effects of a short term fructose diet in young and adult rats, in order to highlight potential age-dependent responses.

One of the main evidence arising from the here presented results is that a short-term high fructose intake induces a condition of low-grade metabolic inflammation in liver of young and adult rats.

Plasma levels of haptoglobin, a reliable marker of inflammation (Lisi et al., 2011; Maffei et al., 2016), as well as hepatic TNF-alpha content and MPO activity increased in both young and adult rats fed a fructose-rich diet. A more detailed analysis showed only slight changes in mRNA expression of macrophage activation markers (mcp1 and arg) and a significant increase in hepatic LCN-2 content in young rats. However, this increase in hepatic LCN-2 might be too early to produce a similar alteration in plasma levels of this inflammation marker, although the liver is considered the main source of circulating LCN-2 (Alwahsh et al., 2014). Interestingly, increased hepatic LCN-2 content together with unchanged LCN-2 plasma levels have been also found in severely obese women (Auguet et al., 2013). In adult rats, no variation was found in hepatic LCN-2 content, but the mRNA expression of markers of macrophage activation drastically increased. Although the data on mRNA in young rats are indicative of a minor activation of macrophages, several other markers of low-grade metabolic inflammation (TNF-alpha, MPO, lipocalin, haptoglobin) were found all increased. This result led to the hypothesis that the increase of TNF-alpha and MPO reflects liver cells activation, rather than recruited macrophages. Taken together, these results suggest that different mechanisms underlie the inflammatory response induced in the liver by short term fructose-rich diet in rats of different age. We could speculate that this differential response in young and adult rats could be partly due to the different levels of hepatic LCN-2, which has been identified as a macrophage modulator (Warszawska et al., 2013; Kang et al., 2018).

Hepatic inflammation is long far known to be linked to the onset of insulin resistance (Shoelson et al., 2006). We found the onset of insulin resistance in the liver of young rats after fructose-rich diet, as evidenced by the decreased activation of the downstream effectors of insulin receptor, IRS1 and Akt. The hepatic insulin resistance found in young but not adult rats after a fructose-rich diet is in agreement with the increased ceramide content, since ceramide is a known promoter of insulin resistance (Chaurasia and Summers, 2015). It should be noted that the hepatic content of triglycerides increased only in adult rats, while hepatic ceramide increased only in young rats after fructose feeding. This latter observation is intriguing and in line with the idea that storage of triglycerides could protect cells from excess of fuels, since cellular fatty acids can also be shuttled to other dangerous metabolic pathways, such as the synthesis of ceramide from palmitoyl-CoA and serine, catalyzed by the enzyme serine palmitoyl-CoA transferase (Unger, 2005). When the analysis of glucose homeostasis was extended at the whole-body level, by determining the glucose and insulin response to a glucose load, with the skeletal muscle being the main tissue involved, we found that a short-term fructose-rich diet elicited a decrease in whole body insulin sensitivity in young and adult rats. In addition, in adult but not in young rats, the hyperinsulinemia was associated with hyperglycemia, thus suggesting that there is a progressive decrease of whole body insulin sensitivity with aging. Taken as a whole, the above results suggest that, in rats of different age, the fructose-driven insulin resistance occurs in the various organs, with different severity and timing of onset.

Inflammation and insulin resistance are strictly linked to cellular oxidative damage (Dandona et al., 2004). Lipid oxidative damage was increased by fructose-rich diet, independent of age, both in the whole tissue and in hepatic mitochondria, while an increased level of N-Tyr, as marker of protein oxidative modifications, in hepatic homogenates and mitochondria, was found in young rats. It has been shown that the increase of ceramide is accompanied by the upregulation of inducible nitric oxide synthase (iNOS), thereby enhancing nitric oxide and peroxynitrite formation (Unger, 2005). Therefore, we hypothesize that the increased ceramide in the liver of young rats leads to the upregulation of i-NOS, which in turn increases hepatic N-Tyr levels in these rats. This idea is also supported by the increase of hepatic insulin resistance in young rats after fructose feeding, since it is known that iNOS promotes insulin resistance in the liver (Sansbury and Hill, 2014).

The picture that emerges by these results is a condition of oxidative stress at hepatic level, particularly in young rats. A possible deleterious consequence of the increased lipid and protein oxidative damage induced by fructose intake could be an impairment of mitochondrial function, although our measurements show that this damage is not yet evident at the end of the 2-weeks dietary treatment. However, it is well established that oxidative damage is deleterious for mitochondrial activity and it is therefore conceivable that a longer period of high fructose intake could induce this metabolic impairment in the liver, thus worsening the fructose-induced hepatic damage. It should be noted that energy intake, expressed per unit lean mass, was significantly higher in young rats compared to adults. Thus, it could be conceivable that the liver of young rats had to deal with higher levels of fructose per unit of weight compared to adult rats. This difference could contribute to the higher degree of liver oxidative stress found in young rats.

Differently from the liver, the lack of increase in plasma TBARS, which were found significantly increased after long-term (8 weeks) high fructose intake in adult rats (Cioffi et al., 2017), suggests that systemic oxidative stress occurs later, i.e., between 2 and 8 weeks of high fructose intake. In addition, hepatic content of glycogen and plasma triglycerides are higher in rats fed a fructose-rich diet, independent of age. These latter findings are in line with the stimulation of the synthesis of P-trioses, which in turn stimulates glycogen synthesis and triglyceride production and secretion, due to the increased inflow of fructose in liver cells (Tappy and Lê, 2010, 2012). In agreement, Francini et al. (2010) reported an increase in glycogen stores in the liver of rats given drinking water containing 10% fructose over 3 weeks.

NPRQ values above 1 found at the end of the dietary treatment in adult rats suggest the stimulation of *de novo* lipogenesis, a metabolic pathway typically increased by high fructose feeding. In agreement, hepatic FAS activity was significantly increased by fructose-rich diet only in adult rats. In young rats, *de novo* lipogenesis is not activated probably because of the insulin resistance in this tissue, taking into account that insulin is a well-defined stimulator of this metabolic pathway (Sanders and Griffin, 2016). The increased fructose-driven lipogenesis in turn causes an increase in whole body lipids in adult rats.

Taken together, the present data indicate that, after a short term fructose-rich dietary treatment, young rats do not increase their body lipids but are exposed to hepatic insulin resistance and hepatic oxidative stress, in line with the finding that increased fructose intake may be an important predictor of metabolic risk in young people, independent of weight status (Ambrosini et al., 2013). Overall, these results indicate the need of corrective nutritional interventions for young people and adults as well for the prevention of fructose-induced metabolic alterations.

## Author contributions

RC, LC, and SI: Conceived the study; all the authors designed the experiments; RC, LC, AM, RCa, RCar, and MT: Performed the experiments; RC, LC, AM, RCa, RCar, MT, GL, and SI: Analyzed the data and performed the statistical analyses; RC, LC, and SI: Drafted the manuscript and all authors contributed in the revision, gave final approval for publication and agreed to be accountable for all aspects of the work in ensuring that questions related to the accuracy or integrity of any part of the work are appropriately investigated and resolved.

### Conflict of interest statement

The authors declare that the research was conducted in the absence of any commercial or financial relationships that could be construed as a potential conflict of interest.

## References

[B1] Alwahsh S. M. Dwyer B. J. Forbes S. Thiel D. H. Starkey Lewis P. J. Ramadori G. (2017). Insulin production and resistance in different models of diet-induced obesity and metabolic syndrome. Int. J. Mol. Sci. 18:285. 10.3390/ijms18020285 28134848 PMC5343821

[B2] Alwahsh S. M. Xu M. Seyhan H. A. Ahmad S. Mihm S. Ramadori G.. (2014). Diet high in fructose leads to an overexpression of lipocalin-2 in rat fatty liver. World. J. Gastroenterol. 20, 1807–1821. 10.3748/wjg.v20.i7.1807 24587658 PMC3930979

[B3] Ambrosini G. L. Oddy W. H. Huang R. C. Mori T. A. Beilin L. J. Jebb S. A. (2013). Prospective associations between sugar-sweetened beverage intakes and cardiometabolic risk factors in adolescents. Am. J. Clin. Nutr. 98, 327–334. 10.3945/ajcn.112.051383 23719557 PMC3712546

[B4] Araujo I. C. Andrade R. P. Santos F. Soares E. S. Yokota R. Mostarda C.. (2016). Early developmental exposure to high fructose intake in rats with NaCl stimulation causes cardiac damage. Eur. J. Nutr. 55, 83–91. 10.1007/s00394-014-0826-5 25564432

[B5] Auguet T. Terra X. Quintero Y. Martínez S. Manresa N. Porras J. A.. (2013). Liver lipocalin 2 expression in severely obese women with non alcoholic fatty liver disease. Exp. Clin. Endocrinol. Diabetes 121, 119–124. 10.1055/s-0032-1331696 23426707

[B6] Bray G. A. Popkin B. M. (2013). Calorie-sweetened beverages and fructose: what have we learned 10 years later. Pediatr. Obes. 8, 242–248. 10.1111/j.2047-6310.2013.00171.x 23625798

[B7] Bray G. A. Popkin B. M. (2014). Dietary sugar and body weight: have we reached a crisis in the epidemic of obesity and diabetes? Diab. Care 37, 950–956. 10.2337/dc13-2085 24652725 PMC9514031

[B8] Bremer A. A. Lustig R. H. (2012). Effects of sugar-sweetened beverages on children. Pediatr. Ann. 41, 26–30. 10.3928/00904481-20111209-09 22224718

[B9] Bremer A. A. Mietus-Snyder M. Lustig R. H. (2012). Toward a unifying hypothesis of metabolic syndrome. Pediatrics 129, 557–570. 10.1542/peds.2011-2912 22351884 PMC3289531

[B10] Busserolles J. Rock E. Gueux E. Mazur A. Grolier P. Rayssiguier Y. (2002). Short-term consumption of a high-sucrose diet has a pro-oxidant effect in rats. Br. J. Nutr. 87, 337–342. 10.1079/BJN2002524 12064343

[B11] Campos V. C. Tappy L. (2016). Physiological handling of dietary fructose-containing sugars: implications for health. Int. J. Obes. 40, S6–S11. 10.1038/ijo.2016.8 27001645

[B12] Castro M. C. Francini F. Gagliardino J. J. Massa M. L. (2014). Lipoic acid prevents fructose-induced changes in liver carbohydrate metabolism: role of oxidative stress. Biochim. Biophys. Acta 1840, 1145–1151. 10.1016/j.bbagen.2013.12.005 24361606

[B13] Castro M. C. Francini F. Schinella G. Caldiz C. Zubiría M. G. Gagliardino J. J.. (2012). Apocynin administration prevents the changes induced by a fructose-rich diet on rat liver metabolism and the antioxidant system. Clin. Sci. 123, 681–692. 10.1042/CS20110665 22738259

[B14] Castro M. C. Massa M. L. Arbeláez L. G. Schinella G. Gagliardino J. J. Francini F. (2015). Fructose-induced inflammation, insulin resistance and oxidative stress: a liver pathological triad effectively disrupted by lipoic acid. Life Sci. 137, 1–6. 10.1016/j.lfs.2015.07.010 26188590

[B15] Castro M. C. Massa M. L. Schinella G. Gagliardino J. J. Francini F. (2013). Lipoic acid prevents liver metabolic changes induced by administration of a fructose-rich diet. Biochim. Biophys. Acta 1830, 2226–2232. 10.1016/j.bbagen.2012.10.010 23085069

[B16] Chaurasia B. Summers S. A. (2015). Ceramides – lipotoxic inducers of metabolic disorders. Trends Endocrinol. Metab. 26, 538–550. 10.1016/j.tem.2015.07.006 26412155

[B17] Cigliano L. Spagnuolo M. S. Crescenzo R. Cancelliere R. Iannotta L. Mazzoli A.. (2018). Short-term fructose feeding induces inflammation and oxidative stress in the hippocampus of young and adult rats. Mol. Neurobiol. 55, 2869–2883. 10.1007/s12035-017-0518-2. 28455700

[B18] Cioffi F. Senese R. Lasala P. Ziello A. Mazzoli A. Crescenzo R.. (2017). Fructose-rich diet affects mitochondrial DNA damage and repair in rats. Nutrients 9:323. 10.3390/nu9040323 28338610 PMC5409662

[B19] Crescenzo R. Bianco F. Coppola P. Mazzoli A. Tussellino M. Carotenuto R.. (2014). Fructose supplementation worsens the deleterious effects of short-term high-fat feeding on hepatic steatosis and lipid metabolism in adult rats. Exp. Physiol. 99, 1203–1213. 10.1113/expphysiol.2014.079632 24972835

[B20] Crescenzo R. Bianco F. Falcone I. Coppola P. Dulloo A. G. Liverini G. (2012). Mitochondrial energetic in liver and skeletal muscle after energy restriction in young rats. Br. J. Nutr. 108, 655–665. 10.1017/S0007114511005903 22085624

[B21] Crescenzo R. Bianco F. Falcone I. Coppola P. Liverini G. Iossa S. (2013). Increased hepatic *de novo* lipogenesis and mitochondrial efficiency in a model of obesity induced by diets rich in fructose. Eur. J. Nutr. 52, 537–545. 10.1007/s00394-012-0356-y 22543624

[B22] Crescenzo R. Bianco F. Falcone I. Prisco M. Dulloo A. G. Liverini G.. (2010). Hepatic mitochondrial energetic during catch-up fat after caloric restriction. Metab. 59, 1221–1230. 10.1016/j.metabol.2009.11.015 20045539

[B23] Crescenzo R. Mazzoli A. Di Luccia B. Bianco F. Cancelliere R. Cigliano L. (2017). Dietary fructose causes defective insulin signaling and ceramide accumulation in the liver that can be reversed by gut microbiota modulation. Food Nutr. Res. 61:1331657 10.1080/16546628.2017.1331657 28659742 PMC5475320

[B24] Dandona P. Aljada A. Bandyopadhyay A. (2004). Inflammation: the link between insulin resistance, obesity and diabetes. Trends Immunol. 25, 4–7. 10.1016/j.it.2003.10.013 14698276

[B25] De Marco N. Tussellino M. Carotenuto R. Ronca R. Rizzolio S. Biffo S.. (2017). Eukaryotic initiation factor eIF6 modulates the expression of Kermit 2/XGIPC in IGF- regulated eye development. Dev. Biol. 427, 148–154. 10.1016/j.ydbio.2017.04.017 28472630

[B26] Farah D. Nunes J. Sartori M. Dias D. D. Sirvente R. Silva M. B.. (2016). Exercise training prevents cardiovascular derangements induced by fructose overload in developing rats. PLoS ONE 11:e0167291. 10.1371/journal.pone.0167291 27930685 PMC5145255

[B27] Fernandes M. A. Custódio J. B. Santos M. S. Moreno A. J. Vicente J. A. (2006). Tetrandrine concentrations not affecting oxidative phosphorylation protect rat liver mitochondria from oxidative stress. Mitochondrion 6, 176–185. 10.1016/j.mito.2006.06.002 16890028

[B28] Flohè L. Otting F. (1974). Superoxide dismutase assay. Meth. Enzymol. 105, 93–104. 10.1016/S0076-6879(84)05013-8 6328209

[B29] Folch J. Lees M. Stanley G. H. S. (1957). A simple method for the isolation and purification of total lipids from animal tissues. J. Biol. Chem. 226, 497–510. 13428781

[B30] Ford C. N. Slining M. M. Popkin B. M. (2013). Trends in dietary intake among US 2- to 6-year-old children, 1989-2008. J. Acad. Nutr. Diet. 113, 35–42. 10.1016/j.jand.2012.08.022 23260722 PMC3531045

[B31] Francini F. Castro M. C. Schinella G. García M. E. Maiztegui B. Raschia M. A.. (2010). Changes induced by a fructose-rich diet on hepatic metabolism and the antioxidant system. Life Sci. 86, 965–971. 10.1016/j.lfs.2010.05.005 20470786

[B32] Jin R. Vos M. B. (2015). Fructose and liver function–is this behind nonalcoholic liver disease? Curr. Opin. Clin. Nutr. Metab. Care 18, 490–495. 10.1097/MCO.0000000000000203 26203597

[B33] Kang S. S. Ren Y. Liu C. C. Kurti A. Baker K. E. Bu G.. (2018). Lipocalin-2 protects the brain during inflammatory conditions. Mol. Psyc. 23, 344–350. 10.1038/mp.2016.243 28070126 PMC5503822

[B34] Kim J. J. Shajib M. S. Manocha M. M. Khan W. I. (2012). Investigating intestinal inflammation in DSS-induced model of IBD. J. Vis. Exp. 60:3678 10.3791/3678 PMC3369627 22331082

[B35] Krawisz J. E. Sharon P. Stenson W. F. (1984). Quantitative assay for acute intestinal inflammation based on myeloperoxidase activity. Assessment of inflammation in rat and hamster models. Gastroenterol. 87, 1344–1350. 6092199

[B36] Li Z. Zhao Z. J. Zhu X. O. Ren Q. S. Nie F. F. Gao J. M.. (2012). Differences in iNOS and arginase expression and activity in the macrophages of rats are responsible for the resistance against *T. gondii* infection. PLoS ONE 7:e35834. 10.1371/journal.pone.0035834 22558235 PMC3338469

[B37] Lisi S. Gamucci O. Vottari T. Scabia G. Funicello M. Marchi M.. (2011). Obesity-associated hepatosteatosis and impairment of glucose homeostasis are attenuated by haptoglobin deficiency. Diabetes 60, 2496–2505. 10.2337/db10-1536 21873550 PMC3178294

[B38] Maffei M. Barone I. Scabia G. Santini F. (2016). The Multifaceted haptoglobin in the context of adipose tissue and metabolism. Endocr. Rev. 37, 403–416. 10.1210/er.2016-1009 27337111

[B39] Mock K. Lateef S. Benedito V. A. Tou J. C. (2017). High-fructose corn syrup-55 consumption alters hepatic lipid metabolism and promotes triglyceride accumulation. J. Nutr. Biochem. 39, 32–39. 10.1016/j.jnutbio.2016.09.010 27768909

[B40] Pénicaud L. Ferré P. Assimacopoulos-Jeannet F. Perdereau D. Leturque A. Jeanrenaud B.. (1991). Increased gene expression of lipogenic enzymes and glucose transporter in white adipose tissue of suckling and weaned obese Zucker rats. Biochem. J. 279, 303–308. 10.1042/bj2790303 1681802 PMC1151580

[B41] Roehrig K. L. Allred J. B. (1974). Direct enzymatic procedure for the determination of liver glycogen. Anal. Biochem. 58, 414–421. 10.1016/0003-2697(74)90210-3 4827390

[B42] Sanders F. W. Griffin J. L. (2016). De novo lipogenesis in the liver in health and disease: more than just a shunting yard for glucose. Biol. Rev. Camb. Philos. Soc. 91, 452–468. 10.1111/brv.12178 25740151 PMC4832395

[B43] Sansbury B. E. Hill B. G. (2014). Regulation of obesity and insulin resistance by nitric oxide. Free Rad. Biol. Med. 73, 383–399. 10.1016/j.freeradbiomed.2014.05.016 24878261 PMC4112002

[B44] Sheldon R. D. Padilla J. Jenkins N. T. Laughlin M. H. Rector R. S. (2015). Chronic NOS inhibition accelerates NAFLD progression in an obese rat model. Am. J. Physiol. 308, G540–G549. 10.1152/ajpgi.00247.2014 25573175 PMC4360049

[B45] Shoelson S. E. Lee J. Goldfine A. B. (2006). Inflammation and insulin resistance. J. Clin. Invest. 116, 1793–1801. 10.1172/JCI29069 16823477 PMC1483173

[B46] Sobrecases H. Lê K. A. Bortolotti M. Schneiter P. Ith M. Kreis R.. (2010). Effects of short-term overfeeding with fructose, fat and fructose plus fat on plasma and hepatic lipids in healthy men. Diabetes Metab. 36, 244–246. 10.1016/j.diabet.2010.03.003 20483648

[B47] Spagnuolo M. S. Maresca B. Mollica M. P. Cavaliere G. Cefaliello C. Trinchese G.. (2014). Haptoglobin increases with age in rat hippocampus and modulates Apolipoprotein E mediated cholesterol trafficking in neuroblastoma cell lines. Front. Cell. Neurosci. 8:212. 10.3389/fncel.2014.00212 25140128 PMC4122225

[B48] Tappy L. Lê K. A. (2010). Metabolic effects of fructose and the worldwide increase in obesity. Physiol. Rev. 90, 23–46. 10.1152/physrev.00019.2009 20086073

[B49] Tappy L. Lê K. A. (2012). Does fructose consumption contribute to non-alcoholic fatty liver disease? Clin. Res. Hepatol. Gastroenterol. 36, 554–560. 10.1016/j.clinre.2012.06.005 22795319

[B50] Toop C. R. Muhlhausler B. S. O'Dea K. Gentili S. (2017). Impact of perinatal exposure to sucrose or high fructose corn syrup (HFCS-55) on adiposity and hepatic lipid composition in rat offspring. J. Physiol. 595, 4379–4398. 10.1113/JP274066 28447343 PMC5491864

[B51] Tussellino M. De Marco N. Campanella C. Carotenuto R. (2012). Involvement of the eukaryotic initiation factor 6 and kermit2/gipc2 in Xenopus laevis pronephros formation. Int. J. Dev. Biol. 56, 357–362. 10.1387/ijdb.120009nd 22689378

[B52] Tussellino M. Ronca R. Carotenuto R. Pallotta M. M. Furia M. Capriglione T. (2016). Chlorpyrifos exposure affects fgf8, sox9, and bmp4 expression required for cranial neural crest morphogenesis and chondrogenesis in Xenopus laevis embryos. Environ. Mol. Mutagen. 57, 630–640. 10.1002/em.22057 27669663

[B53] Unger R. H. (2005). Longevity, lipotoxicity and leptin: the adipocyte defense against feasting and famine. Biochimie 87, 57–64. 10.1016/j.biochi.2004.11.014 15733738

[B54] Warszawska J. M. Gawish R. Sharif O. Sigel S. Doninger B. Lakovits K.. (2013). Lipocalin 2 deactivates macrophages and worsens pneumococcal pneumonia outcomes. J. Clin. Invest. 123, 3363–3372. 10.1172/JCI67911 23863624 PMC3726165

